# Clinical significance of serum neuron-specific enolase in gastric adenocarcinoma

**DOI:** 10.1097/MD.0000000000019829

**Published:** 2020-04-17

**Authors:** Hai Luo, Kexin Shen, Hongyan Sun, Ruiqi Li, Zeming Wang, Zhongshi Xie

**Affiliations:** China-Japan Union Hospital of Jilin University, Changchun, Jilin Province, China.

**Keywords:** diagnosis, gastric adenocarcinoma, neuron-specific enolase

## Abstract

As a biomarker, neuron-specific enolase (NSE) has been widely recognized in the diagnosis of benign diseases and malignant tumors. This study aimed to investigate the potential diagnostic value of NSE in patients with gastric adenocarcinoma.

Serum levels of the NSE were compared between 219 patients with gastric adenocarcinoma and 298 healthy individuals, NSE and clinicopathological parameters were analyzed. Meanwhile, to evaluate the diagnostic capability of NSE, the receiver operating characteristic (ROC), and area under curve (AUC) was calculated.

In the present study, the median serum NSE level of the patient group was 20.770 ng/mL, which was higher than that of the control group 15.625 ng/mL (*P* < .05). Serum NSE level in patients group compared with healthy control was statistically significant (*P* < .05). Serum NSE level was associated with pathological tumor-node-metastasis (pTNM) staging, lymph node metastasis, and distant metastasis in patients with gastric adenocarcinoma. Besides, the AUC of NSE in gastric adenocarcinoma was 0.742, which was higher than those of the other 3 markers (0.573–0.644). Besides, the AUC of the combined 4 markers was higher than any individual marker (0.778).

Serum NSE detecting may have good value for diagnosis of gastric adenocarcinoma. Besides, the combination of NSE, CEA, CA19–9, and CA242 performed even better than any single marker. Thus, the combined detection of the 4 tumor markers may be more useful for the diagnosis of gastric adenocarcinoma.

## 1 Introduction

According to the 2018 GLOBOCAN report, the incidence of gastric cancer accounted for 5.7% of the new cases and the mortality rate accounted for 8.2% of the total number of deaths, the incidence of gastric cancer ranks 5th globally, while the mortality rate ranks 3rd.^[1]^ In order to deal with the increased incidence of cancer, the World Health Organization (WHO) has suggested focusing on early detection and standardized treatment of tumors.^[2]^

Neuron-specific enolase (NSE) is known to be a cell-specific isoenzyme of the glycolytic enzyme enolase and was first found in extracts of brain tissue.^[3]^ NSE as a serum biomarker has been widely used in diagnosis of various malignant tumors in clinical, and it has been found to be associated with melanoma, seminoma, renal cell carcinoma, Merkel cell tumor, carcinoid tumors, dysgerminomas, and immature teratomas, especially small cell lung cancer (SCLC).^[3]^ It has been reported that the NSE was significantly elevated in the serum in patients with SCLC.^[4]^ Although there are some studies on the value of NSE in gastric cancer, the prognostic relationship between NSE and gastric cancer is highly controversial.

This study aimed to analyze the serum NSE for the diagnosis, tumor staging correlation of gastric adenocarcinoma. Meanwhile, we evaluated the diagnostic capacity of combined detection of NSE, CEA, CA19-9, and CA242 for the diagnosis of gastric adenocarcinoma.

## 2 Methods

### 2.1 Patients and samples

This study was approved by Ethics Standards Committee of China-Japan Union Hospital of Jilin University. All patients had signed the informed consent form. All experimental data from the department of Gastrointestinal colorectal surgery, Pathology, Radiology, and Central Research Laboratory of the China-Japan Union Hospital of Jilin University.

Tumors were staged based on tumor invasion (T), lymph node (N), and metastasis (M) classification of the American Joint Committee on Cancer Staging (eighth edition).

The gastric adenocarcinoma group comprised 219 patients who were hospitalized from March 2015 to April 2017 at China-Japan Union Hospital of Jilin University. The patients aged 27 to 87 years. None of the patients received preoperative neoadjuvant chemoradiotherapy or other tumor-related treatment.

All the included patients underwent surgery. Serum samples were collected preoperatively. Patients with stage I–III cancer were treated with radical surgery, while patients at stage IV were given radical surgery or palliative surgery. All patients presented with adenocarcinoma on preoperative pathological examination. All patients had complete clinical and pathological data.

In addition, a control group consisted of 298 healthy volunteers (age range: 20–75 years), who were free of any viral infections or gastrointestinal disease.

### 2.2 Detection of serum tumor markers

Fasting venous blood (2 mL) was taken from the elbow in all patients at 06:00 to 07:00 am on the second day of admission and submitted to the Central Research Laboratory of China-Japan Union Hospital of Jilin University for quantitative analysis of markers. Blood samples were centrifuged at 3500 r/min for 5 minutes, and the supernatant was added to the corresponding tumor kit for detection.

All lab tests were performed in accordance with the standard operating procedures. The experiments were performed on the day of collection, and the reports were used to guide the clinical decisions of the physicians. The cut-off values of the serum markers were in accordance with the recommendations of the manufacturer; specifically 25.00 and 5.00 ng/mL for NSE and CEA, respectively, and 37.00 and 20.00 U/mL for CA19-9 and CA242.

### 2.3 Statistical analysis

Comparisons of the serum levels of the 4 tumor biomarkers between the patients and control groups were evaluated by Mann–Whitney *U* test. The areas under the receiver operating characteristic (ROC) curve (AUC), 95% confidence interval (CI), and Youden index (sensitivity + specificity – 1) were calculated for each tumor biomarker, and the combination of all 4 biomarkers. Logistic regression was conducted to analyze the probability of diagnosing gastric adenocarcinoma using the combination of the 4 biomarkers, the Hosmer-Leme show goodness-of-fit test was used to assess the model. All of the above statistical analyses were performed using SPSS 25.0 (SPSS, Chicago, IL). MedCalc Statistical Software version 15.2.2 (MedCalc Software bvba, Ostend, Belgium; http://www.medcalc.org; 2015) was used to perform *Z* test and compare the AUCs of combined test and single biomarker. *P* < .05 was considered statistically significant.

## 3 Results

### 3.1 Median serum levels of NSE, CEA, CA19-9, and CA242 in healthy controls and patients

The median levels of serum NSE, CEA, CA19-9, CA242 in patients with gastric adenocarcinoma were 20.770, 2.700 ng/mL, 11.840, 6.050 U/mL respectively, comparing with 15.625, 1.765 ng/mL, 9.535, 3.740 U/mL in healthy controls. The serum levels of the 4 markers in the gastric adenocarcinoma patients were significantly higher than the control group (*P* < .05) (Table 1).

**Table 1:**
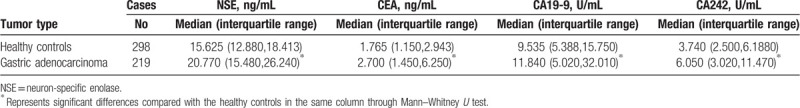
Median serum levels of NSE, CEA, CA19-9, and CA242 in healthy controls and pretreatment patients.

### 3.2 The correlation between NSE level and clinicopathological parameter

Results of the chi-squared tests showed that the serum NSE level was significantly associated with the T, N, M stage or pathological tumor-node-metastasis (pTNM) stage and differentiation, vascular invasion, nerve infiltration, but not with any of the following: sex, or age (Table 2).

**Table 2:**
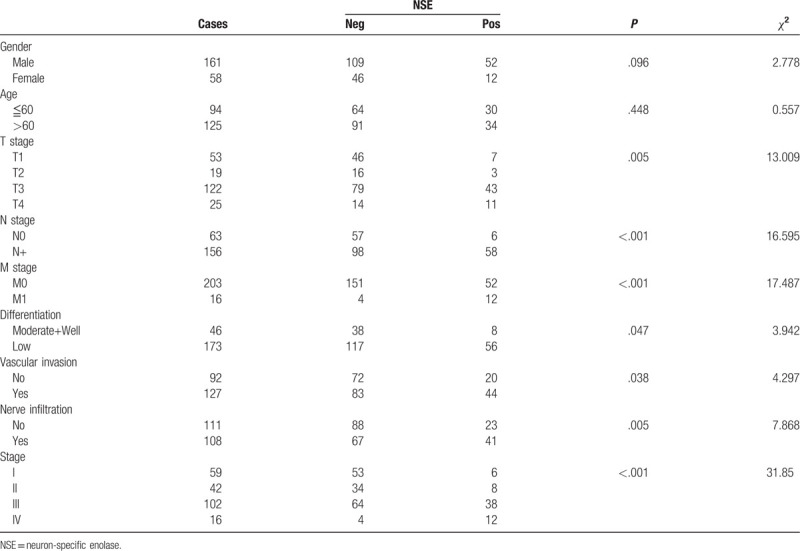
The correlation between NSE level and clinicopathological parameter.

### 3.3 Associations between tumor markers and clinical stage of the disease

**Figure 1:**
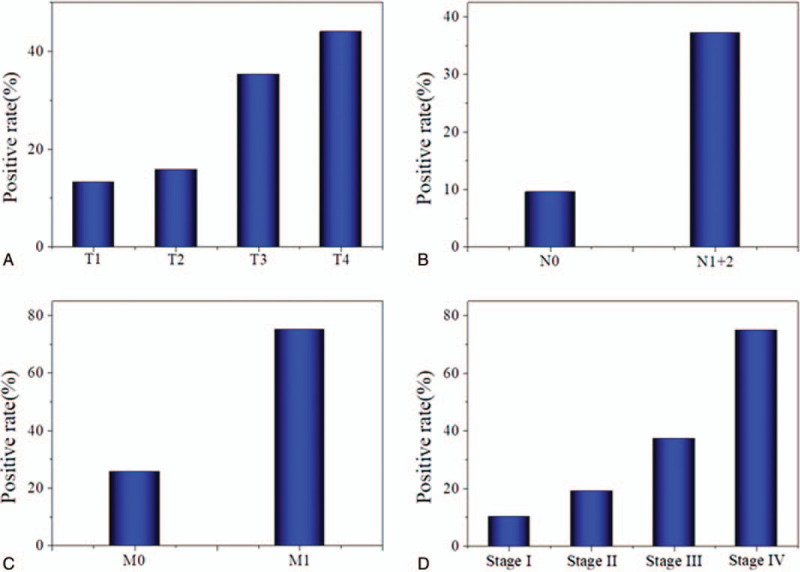
Percentages of patients testing positive for NSE, according to (A) T stage, (B) N stage, (C) N stage, and (D) pTNM stage. NSE = neuron-specific enolase, pTNM = pathological tumor-node-metastasis.

The gastric adenocarcinoma group was stratified by the clinical stages I/II/III/IV, and the positive rates of NSE were calculated (Fig. 1). The rates of positivity of NSE increased with the clinical stage. Also, the rate of positivity of serum NSE was significantly higher in patients with lymph node metastasis or distant metastasis compared with those without.

### 3.4 Logistic regression and receiver operating characteristic curve analyses

**Figure 2:**
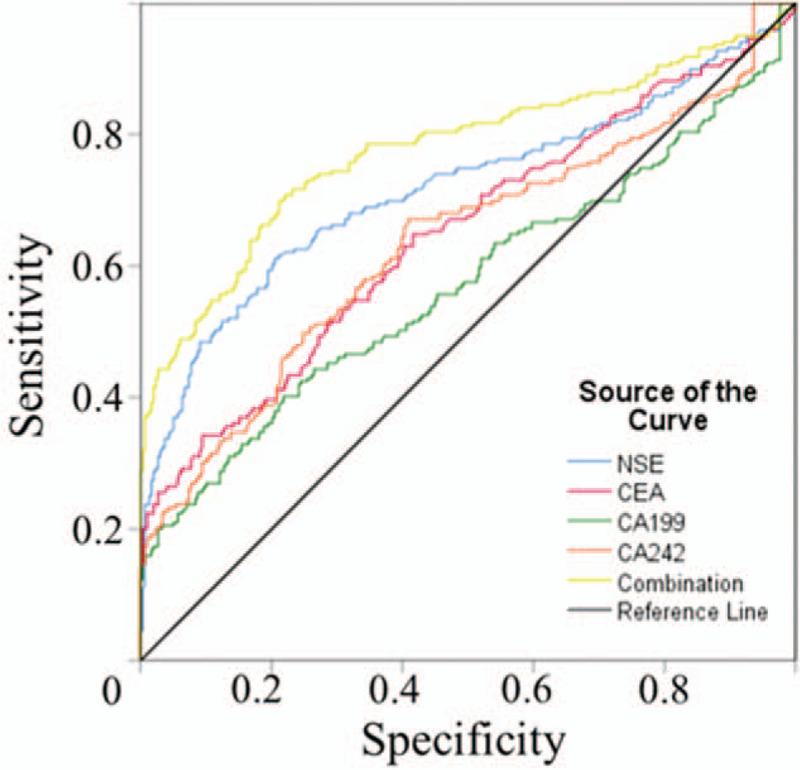
ROC curves of single NSE, CEA, CA19-9, CA242 and the combination in predicting gastric adenocarcinoma. NSE = neuron-specific enolase, ROC = receiver operating characteristic.

For the gastric adenocarcinoma group, ROC curves were constructed for each of the 4 biomarkers, and their combination (Fig. 2). Overall, for the 219 patients, the areas under the ROC curves (AUC) of each marker were as follows: NSE, 0.742; CEA, 0.644; CA19-9, 0.573, and CA242, 0.653. The AUC for the combination of all 4 markers was 0.778 (Table 3).

**Table 3:**
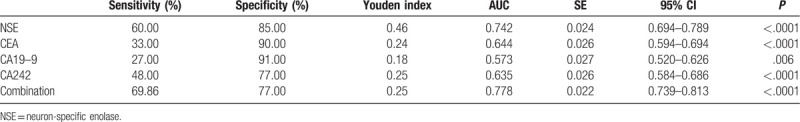
Area under the receiver operating curve (AUC) and the corresponding 95% confidence interval (CI) of the combination of NSE, CEA, CA19-9, and CA242 in gastric cancer.

## 4 Discussion

Serum tumor markers are considered as biological indicators detected from the serum or plasma of suspected tumor patients. The increase of such indicators indicates tumor existence, facilitating pathological analysis, and evaluation of tumor development.^[5]^ Serum tumor biomarkers are useful for choosing treatment strategies, particularly when the markers are convenient and economically efficient to detect.^[6]^ For example, CEA and CA19-9 are often secreted by tumors located in the digestive tract and is the most widely used gastric and colorectal cancers marker.^[7]^ Like the CEA, many cancer biomarkers discovery are eminent in this field due to its anticipated critical role in early diagnosis, therapy guidance, and prognosis monitoring of cancers.^[8]^

NSE is expressed in nerves and cells of neuronal origin.^[9]^ NSE is a well-established tumor marker for SCLC and serum NSE levels are significantly elevated in SCLC patients.^[10]^ But serum NSE levels also higher in patients with non-small cell lung cancer and other types of tumor.^[11]^ The association between serum NSE levels and gastric adenocarcinoma also has been previously reported, but the results were not consistent.^[12,13]^ In the present study, serum NSE levels of gastric adenocarcinoma group and healthy control group were compared and analyzed. The results showed that the serum levels of NSE in the cancer group were significantly higher than those healthy control groups (*P* < .05). Besides, the positive rate of serum NSE in the cancer group (29.15%) is much higher than control group (1.67%). Previous studies have also shown that serum NSE levels in patients with malignancies are markedly increased when compared with healthy persons.^[14]^

According to the 4th edition of WHO digestive tumor classification in 2010 revised nomenclature and classification of neuroendocrine tumors.^[15]^ Neuroendocrine, which has been confirmed to be distributed in adenocarcinoma cells by immunohistochemistry, cannot be included in neuroendocrine tumors. It is named adenocarcinoma together with neuroendocrine cell differentiation (NED) and can still be classified as adenocarcinoma. Therefore, we consider that some neuroendocrine cells may dispersed into adenocarcinoma, which causes an increase in serum NSE in some patients with gastric adenocarcinoma. Relevant studies concerning this are currently limited but are warranted.

We further analyzed the correlation between serum NSE and clinicopathological parameters. The results showed that there was a significant correlation between serum NSE and T stages, N stages, M stages, pTNM stage (*P* < .05). The gastric adenocarcinoma group was stratified by the clinical stages I/II/III/IV, the rates of positivity of NSE increased with the clinical stage, 10.16%, 19.04%, 37.25%, 75.00%, respectively. This indicates that serum NSE level may be as useful as CEA and other markers for the staging of patients with gastric adenocarcinoma, and NSE level may represent a precise indicator of local lymphatic or distant metastasis in gastric adenocarcinoma. NSE may be a good indicator for evaluating the prognosis of gastric adenocarcinoma.

As Liu et al^[16]^ reported, pretreatment elevated CEA and positive MRI-predicted lymph nodes staging-(mrN) are independent risk factors for synchronous distant metastasis in rectal cancer and a combination of both could help to recognize the patients with high risk for structuring personalized treatment protocol. We consider that the evaluation of preoperative synchronous metastasis by NSE combined with imaging examination may be also of great significance, which requires further experiments and follow-up to demonstrate.

The reasons behind the differences in the NSE values between cancer patients in earlier and later clinical stages are not fully understood. It seems likely that the NSE level is closely related to and may reflect the rate of tumor growth. Enolase is a cytoplasmic enzyme that catalyzes the conversion of 2-phosphoglycerate to phosphoenolpyruvate in the glycolytic pathway. In a setting of tumor growth or inflammation, enolase can be released from the cell to control cell growth, immune tolerance, and allergy.^[17]^ The present results warrant further experiments and follow-ups to confirm that NSE is associated with tumor activity.

In the current study, to assess the diagnostic ability of NSE, and other single tumor markers and their combination, ROC curves were constructed and the corresponding AUC were calculated. The AUC of NSE in gastric adenocarcinoma was 0.742, which was higher than the other 3 biomarkers (0.644, 0.573, 0.635, respectively). The sensitivity and specificity of NSE in the diagnosis of CRC were 60.00% and 85.00% respectively. Compared with the other 3 commonly used tumor markers, NSE is relatively reliable for the diagnosis of gastric adenocarcinoma. However, the accuracy of NSE alone for the diagnosis of gastric adenocarcinoma was not satisfactory. Therefore, we also investigated other frequently-used tumor markers: CEA, CA19-9, and CA242. To obtain better diagnostic accuracy, we combined the 4 tumor markers using a logistic regression model. The combined markers in the logistic regression model have improved the diagnostic effect for gastric adenocarcinoma, and the sensitivity of diagnosis was better. This is consistent with previous research.^[18,19]^

This study is limited in that the patients were from a single center, insufficient sample size, and follow-up is lacking. Therefore, NSE as a prognostic indicator in gastric adenocarcinoma remains to be clarified. But we believe that the correlation between NSE and gastric adenocarcinoma will become more clearly in subsequent studies, NSE may be of great value in monitoring recurrence of gastric adenocarcinoma and selecting adjuvant therapy in the foreseeable future.

## 5 Conclusion

This study found that serum NSE detection could be used for gastric adenocarcinoma auxiliary diagnosis. Besides, the combined detection of the tumor markers NSE, CEA, CA19-9, and CA242 is of great significance in the diagnosis of gastric adenocarcinoma.

## Acknowledgments

Thanks to Dr. Sun from Jilin University for providing statistical data analysis.

## Author contributions

Hai Luo participated in the design of the experiment and the analysis of all clinical data as well as the revision of the paper. Kexin Shen participated in the design of the experiment and the analysis of all clinical data as well as the revision of the paper. Hongyan Sun providing all statistical data analysis. Ruiqi Li participated in the collection and analysis of serum markers. Zeming Wang participated in the collection and analysis of imaging data. Zhongshi Xie participated in data analysis and thesis revision. All authors read and approved the final manuscript.
